# Hospital Sunday Fund

**Published:** 1904-05-14

**Authors:** 


### HOSPITAL SUNDAY FUND.

A meeting of the Council of the Metropolitan Hospital Sunday Fund was held at the Mansion House on May 10th, the Lord Mayor being in the chair.

A vote of condolence having been passed to the widow of the late Mr. Hoskin, for many years a member of the Distribution Committee, the secretary, Sir Edmund Hay Currie, read the following letter from Mr. George Herring:—

"Dear Sir E. Hay Currie,—Kindly inform the Council of the Metropolitan Hospital Sunday Fund that I am willing as on previous occasions) to give either £10,000 to the Fund, or to add one quarter to the amount collected in places of worship on June 12th, limiting this to a collection not exceeding £100,000.

"I make this offer with great pleasure, because the Fund is used only for the *maintenance* of hospitals, as I fear that the demands on the charitable for building, etc., may deprive many deserving hospitals of part of their present income and so compel the closing of some of the wards.

"Hoping you will have a record collection,

"I remain, yours truly,

(Signed) "George Herring."

The Secretary stated that during the last five years Mr. Herring had contributed over £53,000 to the Fund.

A cordial vote of thanks to Mr. George Herring for his generous offer was moved by Prebendary Ridgeway and seconded by Prebendary Ingram. It was decided to accept the offer of one-quarter of the amount collected. A vote of thanks having been passed to the Lord Mayor for presiding, the meeting dispersed.

Among those who attended the Council were Sir William Broadbent, Sir William Church, Sir Savill Crossley Sir J. C. Bell, Sir Joseph Savory, Sir George Bruce, Mr. Alfred Willett, F.R.C.S., and others.

